# Effects of monosodium glutamate on testicular structural and functional alterations induced by quinine therapy in rat: An experimental study

**DOI:** 10.18502/ijrm.v19i2.8475

**Published:** 2021-02-21

**Authors:** Davoud Kianifard, Seyyed Maysam Mousavi Shoar, Morteza Fallah Karkan, Ahmed Aly

**Affiliations:** ^1^Department of Basic Sciences, Faculty of Veterinary Medicine, University of Tabriz, Tabriz, Iran.; ^2^Department of Basic Sciences, Faculty of Veterinary Medicine, Shahid Chamran University of Ahvaz, Ahvaz, Iran.; ^3^Department of Urology, Shohada Tajrish Hospital, Shahid Beheshti University of Medical Sciences, Tehran, Iran.; ^4^Beykoz Institute of Life Sciences and Biotechnology, Bezmialem Vakif University, Istanbul 34820, Turkey.

**Keywords:** Male reproductive system, Monosodium glutamate, Quinine hydrochloride, Rat.

## Abstract

**Background:**

Quinine (QU) as an anti-malarial drug induces alterations in testicular tissue. Toxic effects of monosodium glutamate (MSG) on the male reproductive system have been recognized.

**Objective:**

To investigate the impact of MSG administration on the intensity of gonadotoxicity of QU.

**Materials and Methods:**

Sixty eight-wk old Wistar rats weighing 180-200 gr were divided into six groups (n = 10/each): the first group as a control; the second and third groups received low and high doses of MSG (2 & 4 gr/kg i.p.), respectively, for 28 days; the fourth group received QU for seven days (25 mg/kg); and in the fifth and sixth groups, QU was gavaged following the MSG administration (MSG + QU) from day 22 to day 28. Serum testosterone and malondialdehyde (MDA) levels were measured. Testes samples were prepared for tissue MDA levels, histomorphometry, and immunohistochemistry of p53. Sperm analysis was performed on cauda epididymis.

**Results:**

Serum and tissue MDA levels were increased in treated groups compared to the control group. This increment was higher in the MSG + QU groups. The testosterone levels were reduced significantly (p < 0.0001) in all treated groups. In addition, histomorphometric indices and tubular epithelium population were reduced significantly (p < 0.0001) in QU, MSG + QU, and consequently in high-dose MSG, QU, MSG + QU groups. All spermatogenic indices were reduced in the treated groups, particularly in the MSG + QU groups. Sperm motility and viability indices were reduced significantly (p = 0.003) in the MSG + QU groups. Finally, the overexpression of p53 was observed in the MSG + QU groups.

**Conclusion:**

The administration of MSG before and during QU therapy may intensify testicular tissue alterations.

## 1. Introduction 

Quinine (QU) is one of the most common medications used for the treatment of malaria disease due to Plasmodium falciparum. QU treatment is associated with side effects such as irreversible deafness, amblyopia, and at high doses can result in cinchonism syndrome (1).

Some studies have reported QU-induced toxicity on the male reproductive system. Histological alterations of the seminiferous tubules (STD), reduction in testosterone levels, decrease in sperm population and motility, and changes in lipid peroxidation indices have been observed following the administration of QU in experimental animal studies (2, 3).

Monosodium glutamate (MSG), a sodium salt of L-glutamic acid, is the most common food additives that act as a preservative or improves the meal palatability (4). Changing lifestyles and increased access to processed food have led to increased MSG consumption. In this regard, it It has been reported that the average daily intake of MSG through processed foods is about 1 gr in some European countries, 4 gr in Asia, and 10 gr in Germany (5). Some studies have identified the negative toxic effects of MSG on the male reproductive system (6-8). It has been documented that MSG-induced testicular toxicity is associated with increased oxidative stress (4). Oxidative stress can induce the overproduction of reactive oxygen radicals and hydrogen peroxide leading to oxidative DNA damage and peroxidation of cell membranes and consequently cell death (6).

QU as a common antimalarial medication has various side effects on the male reproductive system and MSG is in abundance in many processed foods; these factors can increase the possibility of the simultaneous consumption of these cytotoxic compounds. Accordingly, this study was designed to investigate the potential role of MSG administration prior to QU therapy on the intensity of the QU-induced structural and functional alterations of testicular tissue through the experimental animal study.

## 2. Materials and Methods 

### Chemicals

MSG, QU hydrochloride, EmbryoMax human tubal fluid (HTF medium), and Tris hydrochloride (Tris-HCL) were purchased from Sigma-Aldrich (St Louis, MO 63178, USA). Enzyme immunoassay kit for measuring serum testosterone was obtained from Monobind Inc. USA. Primary antibody (rabbit polyclonal anti p53 antibody) was purchased from St John's Laboratory Ltd., UK. Secondary antibody (goat anti-rabbit IgG) was purchased from Agrisera Antibodies, SE-911 21 Vännäs, Sweden.

### Animals and exposure

In this experimental study, Sixty eight-wk old Wistar rats weighing 180-200 gr were used. The animals were placed in standard cages under a 12-hr light/dark cycle. During the period of the experiment, the standard laboratory chow and water were available ad libitum to the animals.

QU hydrochloride was solubilized in distilled water (20 mg/ml as stock treatment solution) and administered orally (gavage) at a dose of 25 mg/kg once a day for a period of seven days (2). MSG was solubilized in distilled water (concentration: 1 g/ml) and administered intraperitoneally at low- and high doses (2 and 4 gr/kg consequently), respectively, once a day for 28 days (9, 10). The animals were randomly divided into six experimental groups (10 rats/group):

1) Control

2) Low-dose MSG-received (LD-MSG),

3) High-dose MSG-received (HD-MSG),

4) QU-treated (QU),

5) Low-dose MSG+QU (LD-MSG+QU), and

6) High-dose MSG+QU (HD-MSG+QU).

**Figure 1:**
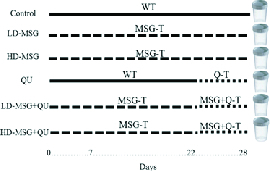
Flowchart representing the animals grouping and treatment. WT: Without treatment; MSG: Monosodium glutamate treatment; Q-T: Quinine treatment; MSG + Q-T: QU was gavaged following the MSG administration.

In group 5 and 6, QU was gavaged following the MSG administration from day 22 to day 28 (Figure 1).

### Biochemical analysis

Twenty-four hours after the final treatment, the animals were anesthetized with xylazine hydrochloride (10 mg/kg i.p.) and ketamine hydrochloride (100 mg/kg i.p.). The blood samples were collected through cardiac puncture. The assessment of the serum testosterone levels was carried out through the standard ELISA method with a commercial assay kit (Monobind Inc. USA).

### Sampling and preparation

The animals were euthanized through sodium thiopental (100 mg/kg i.p.). The left and right testicles were separated and dissected from their epididymis and weighed as total testes weight. Moreover, the organ relative weight (i.e., the organ weight/body weight × 100) was recorded. The left testicles were used for histologic studies and the right testicles were prepared for tissue lipid peroxidation measurement and immunohistochemistry (IHC). The sperm analysis was prepared on the left epididymides.

### Serum and tissue lipid peroxidation levels

The quantification of the serum and tissue lipid peroxidation was completed by the determination of thiobarbituric acid levels (11). Testicles samples were homogenized in 50 mM Tris/HCl, pH 7.5 (1/10, w/v) and centrifuged at 3000 g for 10 min. An aliquot of serum or tissue samples were incubated (95 °C) for 2 hr with thiobarbituric acid. The sample-coated microplates were analyzed using a microplate reader and the absorbance was measured at 532 nm.

### Histomorphometry

Testicular samples were fixed immediately in 10% neutral buffered formalin solution, dehydrated, and paraffin-embedded. The sections were stained with hematoxylin and eosin (H&E) and analyzed with an optical microscope (Olympus CX22, Tokyo, Japan).

The measurement of the height of germinal epithelium (GEH) and the diameter of STD was performed on the images obtained via Am Scope digital camera (Am Scope MD500). The images were processed by the image analysis software (Am Scope, Version ×86, 3.7.7934).

### The population of spermatogenic cells lineage

The number of Sertoli cells, the spermatogonia, the spermatocyte, and the round spermatids were counted in 20 cross-sectioned STD and reported as the mean of the cellular population (total counted cells/20 tubules) for every type of cells (12).

### Microscopic indices of spermatogenesis

The quantitative investigation of spermatogenesis in testicular tissue was completed by the measurement of three indices: tubular differentiation index (TDI, the number of STD with more than three layers of germinal cells derived from type-A spermatogonia); spermiogenesis index (SPI, the ratio of STD with spermatozoids to the empty tubules); and repopulation index (RI, the ratio of active spermatogonia to inactive cells) (13).

### IHC of p53

Paraffin-embedded tissue sections were prepared for immunohistochemical study (14). Briefly, tissue section samples were deparaffinized in xylene and rehydrated through alcohol gradients (90%, 80%, 70%, and 50%). Antigen retrieval was carried out on deparaffinized and rehydrated slides kept in 10 mM sodium citrate solution (pH 6.0) at a temperature of 95°C in a water bath for 40 min. Immunohistochemical staining was conducted according to the manufacturer's protocol (St John's Laboratory Ltd., UK). Endogenous peroxidase activity was blocked with 0.3% H${}_{2}$O${}_{2}$. Tissue slides were washed with PBS (pH 7.2) and incubated with primary antibody (1:500) at 4°C overnight. Sections were treated with horseradish peroxidase (HRP)-conjugated goat anti-rabbit IgG (as secondary antibody) (Agrisera Antibodies, SE-911 21 Vännäs, SWEDEN) at 37°C-incubator humidified chamber container with a wet paper towel for 1 hr. Diaminobenzidine (DAB) chromogen was added to tissue sections and incubated for 5 min. Tissue slides were dehydrated and coverslipped after hematoxylin counterstaining.

### Epididymal sperm analysis

Sperm analysis was conducted on the left epididymides. For sperm counting, the cauda epididymis was cut into small pieces. The contents of epididymis were diluted using the HTF medium (1:200 v:v). After shaking once, 10 µl of the specimen were transferred to a Neubauer hemocytometer and placed for 5 min in the humidified chamber. The number of counted cells in five squares with a light microscope under a 20× microscope objective was expressed as the number of sperm/ ml (15).

For the evaluation of sperm motility percentage, one drop of the specimen was placed on an incubated glass slide (37°C) and covered with a lamella. The percentage of motile cells was recorded in 10 different microscopic fields under a 20× microscope objective.

The evaluation of sperm viability was carried out by adding 10 µl of 0.50% eosin Y and nigrosin staining solution into an equal volume of the specimen. The examination was done on the slides incubated for 2 min at room temperature. The head of dead sperm cells was stained with pink while the head of live cells appeared pale. One hundred randomly chosen spermatozoa were evaluated under a 100× microscope objective.

### Ethical considerations

This study was completed at the Faculty of Veterinary Medicine, University of Tabriz. All animals procedures included in this study were approved by the University of Tabriz Standards for Care and Use of Laboratory Animals (code: IR.TABRIZU.REC.1398.024), in accordance with the Animal Ethical Committee (AEC) of the Ministry of Health and Medical Education of Iran (adopted on April 17, 2006).

### Statistical analysis

Statistical analyses of evaluated parameters were performed using one-way ANOVA followed by Tukey's test. All data are expressed as mean ± SD. Statistical analyses were performed using the GraphPad Prism software (version 5.04; Graph Pad Inc., CA, USA). P < 0.05 was considered as statistically significant.

## 3. Results 

### Weight of testicles

The mean testicular weight was decreased in all treated groups compared to the control group (Table I). This reduction was significant between the control and the HD-MSG + QU groups (p = 0.0119). No significant difference was seen in the organ relative weight between the control and treated groups. (p = 0.344)

### Serum testosterone levels

The blood concentration of testosterone was reduced significantly (p < 0.0001) in all treated groups in comparison to the control group (Table I). The administration of the QU after the MSG consumption led to a reduction in the serum testosterone levels in a dose-dependent manner. Moreover, the administration of the QU following a higher dose consumption of the MSG led to a significant reduction in testosterone levels compared to the groups receiving only LD-MSG or HD-MSG.

### Lipid peroxidation

The levels of malondialdehyde in the serum and testicular tissue were increased in all treated groups compared to the control group (Table I). Treatment of the animals with MSG or QU in individual or combined form led to the dose-dependent increase in serum MDA levels. Accordingly, the most increase in serum MDA levels was observed in the HD-MSG + QU group (Table I). The measurement of MDA levels in testicular tissue showed an increase in this index in all treated groups in comparison to the control group (Table I). The QU-treated group presented a non-significant increase in the tissue MDA levels compared to the control group. However, the animals that received QU or MSG + QU treatment presented the most increase in tissue MDA levels.

### Testicular tissue morphometry

The mean diameter of the STD was reduced in all treated groups compared to the control group (Table I). This reduction was significant in the QU and the MSG + QU treated groups (p < 0.0001). Accordingly, the administration of the QU following the MSG induced a significant reduction in the tubular diameter in comparison to the individual administration of the MSG. The higher dose of the MSG induced more decline in the tubular diameter compared to the lower dose. Accordingly, the height of the GEH was decreased in all treated groups compared to the control group. This reduction was significant (p < 0.0001) in the QU and the MSG + QU groups. The highest reduction in the height of the GEH was observed in the HD-MSG + QU group.

### The population of spermatogenic cells

Table II shows the mean germ cells population. Separate administration of the QU and a higher dose of the MSG led to a significant reduction in the population of GEH cells compared to the control group (p < 0.0001). This significant reduction was also observed in the MSG + QU groups.

### Microscopic indices of spermatogenesis

The mean of all indices of spermatogenesis was reduced non-significantly in MSG-treated groups in comparison to the control group (Table II). The administration of the QU or the LD-MSG + QU led to a significant decrease in the TDI and RI and a non-significant decrease in the SPI compared to the control group. Moreover, the administration of the QU following the higher dose of MSG led to a significant decrease in all indices of spermatogenesis in comparison to the control and the LD-MSG-receiving groups.

### Sperm analysis

All indices of sperm analysis were reduced non-significantly in the MSG-receiving groups in comparison to the control group (Table II). The administration of the QU led to a non-significant decrease in the sperm count and sperm motility indices and a significant decrease in the sperm viability index compared to the control group (p = 0.0003). In addition, compared to the control group, a reduction in the sperm count (non-significant) and sperm viability and motility (significant) was observed in the LD-MSG + QU group. All of these indices were reduced significantly in the HD-MSG + QU group in comparison to the control group.

### Histology of testicular tissue

**Figure 2:**
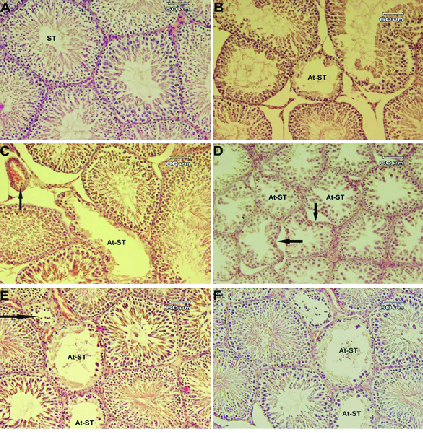
Transverse sections of testicular tissue in experimental groups. (A) Control group, the normal structure of the seminiferous tubules (ST) with regular germinal epithelium columns and narrow interstitial connective tissue. (B) LD-MSG-treated group, atrophied seminiferous tubule (At-ST) with irregular epithelium, the loss of germ cells, and the increase in spacing between tubules. (C) HD-MSG-treated group, atrophied seminiferous tubule (At-ST) with dissociation of germ cells and arterial hyperemia (black arrow). (D) QU-treated group, atrophied seminiferous tubules (At-ST) disarrangement of tubular architecture (black arrows), and loss of post meiosis cells. (E) LD-MSG + QU-treated group, tubular dissociation (black arrow) with the arrest of spermatogenesis. (F) HD-MSG + QU-treated group, sever tubular depletion with spermatogenesis arrest, and negative microscopic indices of spermatogenesis. H&E staining, 200×.

The histologic study showed various alterations in testicular tissue of treated groups in comparison to the control group (Figure 2). The atrophy of STD and loss of tubular architecture, a decrease in the germ cells population, impaired cellular arrangement, and the increase in the interstitial connective tissue were the most prominent alterations observed in the treated groups. These changes were observed in higher degrees in the MSG + QU groups.

### IHC of p53

**Figure 3:**
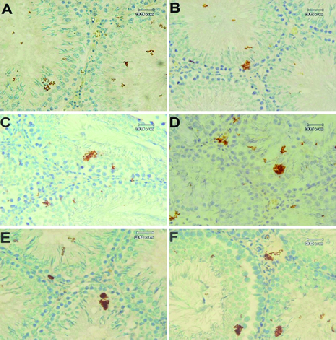
Immunohistochemical staining of testicular tissue for p53 in experimental groups. (A) Control group, faint positive reaction to p53 expression (brown chromogen sites). (B) LD-MSG-treated group, the expression of p53 has been increased compared to the control group. (C) HD-MSG-treated group, increased positive reaction to p53 expression compared to the control and LD-MSG-treated groups. (D) QU-treated group, the expression of p53 has been observed in more degrees. (E) LD-MSG + QU-treated group, the expression of p53 positive areas has been increased in comparison to the control, MSG, or QU-treated group. (F) HD-MSG + QU-treated group, the overexpression of p53 has been observed compared to the control and other treated groups. 400×.

Immunostaining of testicular tissue for the detection of the p53 expression showed the increase in the positive reaction areas to p53 expression in treated groups in comparison to the control group (Figure 3). The expression of p53 was higher in the QU-treated group compared to the MSG-treated groups. Moreover, tissue samples of the MSG + QU-treated groups showed a higher reaction to p53 expression compared to the control and other treated groups.

**Table 1:** Mean of the testes weight, MDA levels, testosterone levels, and testicular tissue morphometry in experimental groups


	**Control**	**LD-MSG**	**HD-MSG**	**QU**	**LD-MSG + QU**	**HD-MSG + QU**	**P-value**
**Testes weight (g)**	2.025 ± 0.1666	1.956 ± 0.1713	1.970 ± 0.2642	1.709 ± 0.1860	1.861 ± 0.3397	1.636 ± 0.2627${}^{a}$	0.011
**Relative organ weight (%)**	0.80 ± 0.083	0.75 ± 0.095	0.78 ± 0.085	0.72 ± 0.076	0.73 ± 0.091	0.71 ± 0.124	0.344
**Serum MDA (nmol.mg protein)**	0.81 ± 0.21	1.01 ± 0.28	1.41 ± 0.41	1.37 ± 0.57	1.22 ± 0.51	1.85 ± 0.58${}^{ab}$	0.0011
**Tissue MDA (nmol.mg protein)**	0.87 ± 0.21	1.15 ± 0.29	1.09 ± 0.26	1.40 ± 0.42	1.32 ± 0.47	1.71 ± 0.48${}^{ac}$	0.0016
**Testosterone (ng/ml)**	0.451 ± 0.10${}^{a}$	0.327 ± 0.09${}^{a}$	0.325 ± 0.12${}^{a}$	0.218 ± 0.06${}^{a}$	0.226 ± 0.05${}^{a}$	0.198 ± 0.03${}^{abc}$	<0.0001
**ST Diameter (µm)**	265.6 ± 20.94	247.5 ± 18.50	236.2 ± 28.77	212.4 ± 18.72${}^{ab}$	201.4 ± 31.64${}^{abc}$	197.1 ± 27.65${}^{abc}$	<0.0001
**GE Height (µm)**	101.8 ± 13.99	85.38 ± 19.37	81.38 ± 14.37	71.13 ± 16.20${}^{a}$	71.88 ± 12.36${}^{a}$	58.88 ± 10.23${}^{abc}$	<0.0001
Data are expressed as Mean ± SD. One-way ANOVA test. ${}^{a}$P < 0.05 compared to the control group, ${}^{b}$P < 0.05 compared to the LD-MSG group, ${}^{c}$P < 0.05 compared to the HD-MSG group. Relative organ weight (%): Organ (Testes) to body weight ratio, LD-MSG: Low-dose monosodium glutamate, HD-MSG: High-dose monosodium glutamate, QU: Quinine, ST: Seminiferous tubule, GE: Germinal epithelium							

**Table 2:** Mean of the germ cells population, the microscopic indices of spermatogenesis, and the sperm analysis indices in experimental groups


	**Control**	**LD-MSG**	**HD-MSG**	**QU**	**LD-MSG + QU**	**HD-MSG + QU**	**P-value**
**Sertoli (mean of cells/20 tubules) **	22.63 ± 2.06	20.13 ± 2.58	18.25 ± 1.98${}^{a}$	15.00 ± 3.66${}^{ab}$	14.13 ± 3.22${}^{abc}$	13.63 ± 2.26${}^{abc}$	<0.0001
**Spermatogonia (mean of cells/20 tubules)**	60.88 ± 9.55	51.75 ± 8.03	45.88 ± 3.39${}^{a}$	47.88 ± 8.77${}^{a}$	43.38 ± 5.97${}^{a}$	39.25 ± 7.20${}^{ab}$	<0.0001
**Spermatocyte (mean of cells/20 tubules)**	70.88 ± 11.78	62.13 ± 7.18	52.75 ± 9.82${}^{a}$	53.00 ± 8.36${}^{a}$	47.63 ± 5.73${}^{ab}$	41.00 ± 6.90${}^{ab}$	<0.0001
**Spermatid (mean of cells/20 tubules)**	278.9 ± 18.38	249.8 ± 35.64	163.9 ± 23.79${}^{ab}$	149.0 ± 16.73${}^{ab}$	137.3 ± 23.55${}^{ab}$	131.8 ± 18.43${}^{ab}$	<0.0001
**TDI (%)**	72.25 ± 9.17	63.63 ± 6.14	59.88 ± 10.40	52.88 ± 13.34${}^{a}$	50.88 ± 6.12${}^{a}$	47.50 ± 7.36${}^{ab}$	<0.0001
**SPI (%)**	63.25 ± 6.274	61.13 ± 7.473	59.00 ± 10.90	51.25 ± 7.025	51.38 ± 9.899	47.38 ± 6.368${}^{ab}$	0.0013
**RI (%)**	73.63 ± 7.170	67.75 ± 5.849	63.00 ± 8.864	61.25 ± 7.440${}^{a}$	59.63 ± 8.348${}^{a}$	53.13 ± 6.175${}^{ab}$	<0.0001
**Sperm count (10${}^{6}$ mL${}^{-1}$)**	26.63 ± 5.65	22.50 ± 4.62	24.00 ± 4.59	19.38 ± 4.74	20.75 ± 6.25	17.00 ± 3.16${}^{a}$	0.0058
**Sperm viability (%)**	64.75 ± 4.92	58.75 ± 8.44	55.88 ± 7.49	49.63 ± 8.74${}^{a}$	52.00 ± 7.03${}^{a}$	48.00 ± 6.52${}^{a}$	0.0030
**Sperm motility (%)**	60.13 ± 5.592	57.25 ± 6.840	53.00 ± 8.298	52.63 ± 7.520	47.13 ± 5.222${}^{a}$	47.25 ± 8.598${}^{a}$	0.0030
Data are expressed as Mean ± SD. One-way ANOVA test. ${}^{a}$P < 0.05 compared to the control group, ${}^{b}$P < 0.05 compared to the LD-MSG group,${}^{c}$P < 0.05 compared to the HD-MSG group. LD-MSG: Low-dose monosodium glutamate, HD-MSG: High-dose monosodium glutamate, QU: Quinine							

## 4. Discussion

In this study, the role of the administration of MSG (as a common food additive with toxic properties) on the intensity of testicular tissue structural and functional changes related to QU therapy was investigated. This study showed that the administration of MSG prior to QU therapy resulted in more alterations of testicular tissue induced by QU in adult rats.

A wide range of medications is used to treat malaria disease caused by *P. falciparum*. QU is one of the most commonly used drugs in the treatment of malaria. However, some studies have pointed out the side effects of this drug including neurotoxicity, cardiotoxicity, and testicular toxicity (1-3, 16). MSG is a common food additive found in processed foods that acts as a preservative to improve the palatability of meal. This compound is available to a variety of age groups through a wide range of foods. Consumption of MSG is associated with alterations in the structure and the function of testicular tissue (6-8). There are several roles for glutamate in biological systems such as gene expression regulation, antioxidant reaction, and immune responses (17). Some MSG-related side effects such as brain neurotoxicity, obesity and metabolic defects, Chinese restaurant syndrome, and the negative effects on reproductive organs have been discussed more (18).

In this study, the mean testicular weight was decreased in the experimental groups compared to the control group. This reduction was observed in higher degrees in the QU-treated and the HD-MSG + QU-treated groups. The measurement of absolute and relative organ weight is an important index in the toxicological studies (19). Moreover, it has been demonstrated that the weight of testes depends on the mass of differentiated spermatogenic cells (20). According to the results of testicular weight and histomorphometry, we can suggest that the administration of the QU through induction of the loss of germ cells population might be involved in the reduction of testicular weight. It seems that the administration of QU following the MSG administration has more negative effects on the weight of testes.

This study demonstrated that the administration of the MSG or the QU led to a significant reduction in the blood testosterone levels. This reduction was seen more once the QU was gavaged to rats after MSG consumption. The reduction of testosterone levels has been reported in the QU-treated rats (2, 21). Also, some studies have pointed out the effects of MSG on testosterone reduction (7, 22). Decrease in the testosterone levels may be related to the functional alterations of testicular tissue due to increased oxidative stress and probably a change in the population of Leydig cell. Moreover, decreased testosterone levels may be due to seminiferous tubular damage and subsequently decrease in the diameter of tubules (3). In the present study, a decrease in testosterone levels was consistent with a decrease in histomorphometric indices of testicular tissue so that the groups that received both the MSG and the QU showed the greatest decrease in measured structural and functional indices compared to the groups that received the MSG or the QU alone. Based on the significant decrease in the Sertoli cells population and testosterone levels in the HD-MSG + QU-receiving group compared to the MSG-receiving groups, it could be proposed that the administration of MSG before and during the QU therapy could involve in exacerbation of the structural changes in the testicular tissue through diminished spermatogenesis process and subsequent decrease of tubular cellularity. The microscopic indices of spermatogenesis (as an indicator for cell proliferation) were reduced more in the MSG + QU-receiving groups in comparison to the MSG- or QU-treated groups. So, given the role of testosterone in spermatogenesis (23), it can be established that the administration of MSG before a QU therapy could decrease spermatogenesis through testicular dysfunction in testosterone production.

Testicular tissue due to its cellular characteristics is susceptible to environmental factors. Accordingly, many xenobiotic toxicants such as chemicals and drugs could influence the alterations in the testicular microenvironment (24). It has been reported that the testicular tissue damages related to the administration of MSG or QU have a close association with the reactive oxygen species (ROS) overproduction (2, 4, 25). The results of this study showed an increase in the serum and tissue MDA levels (as one of the final products of lipid peroxidation) in all treated groups. These results are in agreement with previous reports indicating the increase in ROS in the testicular tissue following the administration of the MSG or QU (2-4). It was also found that the highest increase in the MDA levels occurs in the HD-MSG + QU group. This result indicates that the administration of the MSG prior to a QU therapy can induce more cellular and tissue damage associated to oxidative stress compared to distinct QU treatment of animals.

Testicular tissue due to its considerable amount of lipids is susceptible to peroxidation. Various studies have demonstrated the cellular and tissue alterations of the STD following the administration of MSG or QU (2, 4, 8, 26). In this study, the results obtained by the histologic and morphometric evaluations showed prominent changes in the STD in treated groups. These changes included cellular destruction, impaired tubular architecture, and spermatogenic alterations that are closely related to cell population decline. Some studies have suggested the presence of glutamate receptors in various tissues, such as the hypothalamus, spleen, thymus, liver, kidneys, endocrine system, ovaries, and testes (27, 28). Therefore, considering the presence of glutamate transporters and receptors in testicular tissue and the function of MSG in increasing tissue oxidative stress, it seems reasonable to speculate that the cellular damages, loss of cellular population, and decrease in spermatogenesis have a direct association with the negative effects of glutamate on tubular GEH through an increase in the ROS-related lipid peroxidation in developing germ cells in the MSG-receiving groups. However, the important point of the present study was that the alterations of the microscopic structure of testes were observed more once the QU therapy was performed following the MSG consumption.

Moreover, ROS play an essential role in the normal function of spermatozoa through the stimulation of sperm capacitation, acrosome reaction, and oocyte fusion (29). However, due to high levels of structural lipids in spermatozoa, the increased levels of ROS production can induce structural and functional damage. In this study, we verified a decrease in the sperm analysis indices in the MSG- or the QU-treated groups. Our results are consistent with previous studies that have reported a reduction in sperm analysis indices following the administration of QU or MSG (2, 4). Moreover, the results of the present study showed a significant decrease in sperm count, sperm viability, and motility in the HD-MSG + QU-receiving group. Furthermore, the results of testicular histomorphometry and sperm analysis indicate the intensification of changes in the MSG + QU-treated groups in comparison to the individual QU therapy. Although a part of the observed changes are related to the toxic effects of QU on spermatozoa, the administration of MSG before the QU therapy exacerbated the sperm parameter alterations related to QU therapy. Besides, overproduction of ROS could induce various damages in the cellular membrane of spermatozoa that can lead to peroxidation of plasma membrane lipids and subsequently cellular structural abnormalities (30). Accordingly, increased production of ROS due to QU therapy and its integration with ROS overproduction induced by the MSG may be one of the mechanisms involved in the damage of germ cell lineage and decrease in the sperm quantity and quality as well.

Tumor protein p53 is one of the regulatory proteins involved in the cell cycle through the activation of DNA repair proteins or initiation of apoptosis after severe DNA damage. The p53 is often upregulated after DNA damage resulting in the initiation of apoptosis (31, 32). In the physiological conditions, apoptosis occurs in 20% of developing germ cells. However, an increase in apoptosis could induce some alterations in germ cell lineage and sperm parameters (33). Consequently, the expression level of this protein may indicate the amount of oxidative stress-induced cellular damage. In this study, the immunostaining of testicular tissue showed that the consumption of MSG prior to the QU treatment induced more upregulation of p53 compared to the individual QU therapy. Moreover, the most positive areas to the p53 expression were observed in the adluminal compartment of the STD which is the location of the round spermatids and spermatozoids. This finding suggests that the round spermatids and developing spermatozoids are more sensitive compared to other germ cells when exposed to chemical stressors. Accordingly, the consistency of the immunohistochemical results with the histomorphometric results indicates that the ROS overproduction related to the treatment with QU after a period of MSG consumption induces DNA damage in developing germ cells through the induction of p53 upregulation and the initiation of the apoptotic pathways.

Based on the results of previous studies and the results of this experiment, it can be established that the main mechanism for tissue alterations following the administration of MSG or QU is related to increased oxidative stress. So, it can be suggested that the administration of MSG before and during QU therapy increases the structural and functional changes in testicular tissue by increasing the amount of ROS and its related cellular and tissues alterations.

## 5. Conclusion 

This study showed that in adult rats, the administration of MSG prior to QU therapy increases the gonadotoxic effects of this antimalarial drug. This study indicates a potential role of MSG (as a common dietary supplement with cytotoxic and organotoxic characteristics) in increasing the QU-induced alterations in the male reproductive system. However, further studies are required to explain the mechanisms involved in the incidence of the aforementioned changes.

##  Conflict of Interest

There is no conflict of interest.

## References

[1] Kremsner PG, Zotter GM, Bach M, Graninger W. A case of transient organic brain syndrome during quinine treatment. *Rev Soc Bras Med Trop* 1989; 22: 53. 10.1590/s0037-86821989000100010 2638024

[2] Izaguirry AP, Pavin NF, Soares MB, Spiazzi CC, Araújo FA, Michels LR, et al. Effect of quinine-loaded polysorbate-coated nanocapsules on male and female reproductive systems of rats. *Toxicol Res* 2016; 5: 1561–1572. 10.1039/c6tx00203j PMC6061987 30090457

[3] Farombi EO, Ekor M, Adedara IA, Tonwe KE, Ojujoh TO, Oyeyemi MO. Quercetin protects against testicular toxicity induced by chronic administration of therapeutic dose of quinine sulfate in rats. *J Basic Clin Physiol Pharmacol* 2012; 23: 39–44. 10.1515/jbcpp-2011-0029 22865448

[4] Hamza RZ, Al-Harbi MS. Monosodium glutamate induced testicular toxicity and the possible ameliorative role of vitamin E or selenium in male rats. *Toxicol Rep* 2014; 22: 1037–1045. 10.1016/j.toxrep.2014.10.002 PMC5598534 28962317

[5] Park E, Yu KH, Kim DK, Kim S, Sapkota K, Kim SJ, et al. Protective effects of N-acetyl cysteine against monosodium glutamate induced astrocytic cell death. *Food Chem Toxicol *2014; 67: 1–9. 10.1016/j.fct.2014.02.015 24556569

[6] Rahimi Anbarkeh F, Baradaran R, Ghandy N, Jalali M, Nikravesh MR, Soukhtanloo M. Effects of monosodium glutamate on apoptosis of germ cells in testicular tissue of adult rat: An experimental study. *Int J Reprod Biomed* 2019; 17: 261–270. 10.18502/ijrm.v17i4.4551 PMC6686650 31435603

[7] Igwebuike UM, Ochiogu IS, Ihedinihu BC, Ikokide JE, Idika IK. The effects of oral administration of monosodium glutamate (MSG) on the testicular morphology and cauda epididymal sperm reserves of young and adult male rats. *Vet Arch* 2011; 81: 525–534.

[8] Alalwani AD. Monosodium glutamate induced testicular lesions in rats (histological study). *Middle East Fertil Soc J* 2014; 19: 274–280.

[9] Calis IU, Cosan DT, Saydam F, Kolac UK, Soyocak A, Kurt H, et al. The effects of monosodium glutamate and tannic acid on adult rats. *Iran Red Crescent Med J* 2016; 18: e37912.

[10] Pavlovic V, Pavlovic D, Kocic G, Sokolovic D, Sarac M, Jovic Z. Ascorbic acid modulates monosodium glutamate induced cytotoxicity in rat thymus. *Bratisl Lek Listy *2009; 110: 205–209. 19507646

[11] Bahrami S, Shahriari A, Tavalla M, Azadmanesh S, Hamidinejat H. Blood levels of oxidant/antioxidant parameters in rats infected with toxoplasma gondii. *Oxid Med Cell Longev* 2016; 2016: 8045969. 10.1155/2016/8045969 PMC5056297 27746857

[12] Miresmaeili SM, Halvaei I, Fesahat F, Fallah A, Nikonahad N, Taherinejad M. Evaluating the role of silver nanoparticles on acrosomal reaction and spermatogenic cells in rat. *Iran J Reprod Med* 2013; 11: 423–430. PMC3941417 24639775

[13] Gholirad S, Razi M, Hassani Bafrani H. Tracing of zinc and iron in experimentally induced varicocele: Correlation with oxidative, nitrosative and carbonyl stress. *Andrologia* 2017; 49: 1–11. 10.1111/and.12687 27682184

[14] Mazroa SA, Asker SA, Ellatif MA, Asker W. Microscopic structural alterations and p53 immune-expression in adult rat testis in response to intra-tunical versus intra-parenchymal injection of methylene blue. *Int J Clin Exp Pathol* 2016; 9: 5346–5356.

[15] Lucio RA, Tlachi-Lopez JL, Eguibar JR, Agmo A. Sperm count and sperm motility decrease in old rats. *Physiol Behav* 2013; 110–111: 73–79. 10.1016/j.physbeh.2012.12.015 23296084

[16] White NJ. Cardiotoxicity of antimalarial drugs. *Lancet Infect Dis* 2007; 7: 549–558. 10.1016/S1473-3099(07)70187-1 17646028

[17] Wu G. Functional amino acids in growth, reproduction, and health. *Adv Nutr* 2010; 1: 31–37. 10.3945/an.110.1008 PMC3042786 22043449

[18] Husarova V, Ostatnikova D. Monosodium glutamate toxic effects and their implications for human intake: A review. *J Med Res* 2013; 2013: 608765.

[19] Chinta G, Coumar MS, Periyasamy L. Reversible testicular toxicity of piperine on male albino rats. *Pharmacogn Mag* 2017; 13 (Suppl.): S525–S532. 10.4103/pm.pm_405_16 PMC5669092 29142409

[20] Katoh C, Kitajima S, Saga Y, Kanno J, Horii I, Inoue T. Assessment of quantitative dual-parameter flowcytometric analysis for the evaluation of testicular toxicity using cyclophosphamide- and ethinylestradiol-treated rats. *J Toxicol Sci* 2002; 27: 87–96. 10.2131/jts.27.87 12058451

[21] Osinubi AA, Ajala MO, Noronha CC, Okanlawon AO. Quinine lowers serum and testicular testosterone in adult Sprague-Dawley rats. *Afr J Med Med Sci* 2006; 35: 425–430. 17722807

[22] Iamsaard S, Sukhorum W, Samrid R, Yimdee J, Kanla P, Chaisiwamongkol K, et al. The sensitivity of male rat reproductive organs to monosodium glutamate. *Acta Med Acad* 2014; 43: 3–9. 10.5644/ama2006-124.94 24893633

[23] Ramaswamy S, Weinbauer GF. Endocrine control of spermatogenesis: Role of FSH and LH/ testosterone. *Spermatogenesis* 2014; 4: e996025. 10.1080/21565562.2014.996025 PMC4581062 26413400

[24] Cheng CY, Wong EWP, Lie PPY, Li MWM, Su L, Siu ER, et al. Environmental toxicants and male reproductive function. *Spermatogenesis* 2011; 1: 2–13. 10.4161/spmg.1.1.13971 PMC3158642 21866273

[25] Krishnaveni M, Suresh K. Induction of apoptosis by quinine in human laryngeal carcinoma cell line (KB). *Int J Curr Res Aca Rev* 2015; 3: 169–178.

[26] Osinubi AA, Noronha CC, Okanlawon AO. Morphometric and stereological assessment of the effects of long-term administration of quinine on the morphology of rat testis. *West Afr J Med* 2005; 24: 200–205. 10.4314/wajm.v24i3.28198 16276694

[27] Mohamed AA, Thabet H, Abdel-hafez AM. Toxicity of monosodium glutamate on male rat reproductive system and effect of curcumin and propolis co-administeration. *Egypt J Forensic Sci Appl Toxicol* 2017; 17: 129–146.

[28] Jubaidi FF, Mathialagan RD, Noor MM, Taib IS, Budin SB. Monosodium glutamate daily oral supplementation: Study of its effects on male reproductive system on rat model. *Syst Biol Reprod Med* 2019; 65: 194–204. 10.1080/19396368.2019.1573274 30773941

[29] Wu PY, Scarlata E, O'Flaherty C. Long-term adverse effects of oxidative stress on rat epididymis and spermatozoa. *Antioxidants* 2020; 9: 170. 10.3390/antiox9020170 PMC7070312 32093059

[30] Gozukara KH, Ozcan O, Ozgur T, Kaya YS, Tutuk O. Protective effects of colchicine on testi- cular torsion/detorsion-induced ischemia/reperfus- ion injury in rats. *Urol J* 2020; 17: 294–300. 10.22037/uj.v0i0.4918 31364099

[31] Levine AJ, Puzio-Kuter AM. The role of p53 in metabolic regulation. *Genes Cancer* 2011; 2: 385–391. 10.1177/1947601911409738 PMC3135642 21779507

[32] Li M, He Y, Dubois W, Wu X, Shi J, Huang J. Distinct regulatory mechanisms and functions for p53- activated and p53-repressed DNA damage response genes in embryonic stem cells. *Mol Cell* 2012; 46: 30–42. 10.1016/j.molcel.2012.01.020 PMC3327774 22387025

[33] Nirupama M, Devaki M, Nirupama R, Yajurvedi HN. Chronic intermittent stress-induced alterations in the spermatogenesis and antioxidant status of the testis are irreversible in albino rat. *J Physiol Biochem* 2013; 69: 59–68. 10.1007/s13105-012-0187-6 22820994

